# Potential association of genetically predicted lipid and lipid-modifying drugs with rheumatoid arthritis: A Mendelian randomization study

**DOI:** 10.1371/journal.pone.0298629

**Published:** 2024-02-28

**Authors:** Zhican Huang, Ting Cui, Jin Yao, Yutong Wu, Jun Zhu, Xin Yang, Li Cui, Haiyan Zhou

**Affiliations:** 1 School of Acupuncture and Tuina, Chengdu University of Traditional Chinese Medicine, Chengdu, China; 2 School of Health and Rehabilitation, Chengdu University of Traditional Chinese Medicine, Chengdu, China; Graduate School of Medicine, Kyoto University, JAPAN

## Abstract

**Background:**

Past studies have demonstrated that patients diagnosed with rheumatoid arthritis (RA) often exhibit abnormal levels of lipids. Furthermore, certain lipid-modifying medications have shown effectiveness in alleviating clinical symptoms associated with RA. However, the current understanding of the causal relationship between lipids, lipid-modifying medications, and the risk of developing RA remains inconclusive. This study employed Mendelian randomization (MR) to investigate the causal connection between lipids, lipid-modifying drugs, and the occurrence of RA.

**Methods:**

We obtained genetic variation for lipid traits and drug targets related to lipid modification from three sources: the Global Lipids Genetics Consortium (GLGC), UK Biobank, and Nightingale Health 2020. The genetic data for RA were acquired from two comprehensive meta-analyses and the R8 of FINNGEN, respectively. These variants were employed in drug-target MR analyses to establish a causal relationship between genetically predicted lipid-modifying drug targets and the risk of RA. For suggestive lipid-modified drug targets, we conducted Summary-data-based Mendelian Randomization (SMR) analyses and using expression quantitative trait loci (eQTL) data in relevant tissues. In addition, we performed co-localization analyses to assess genetic confounders.

**Results:**

Our analysis revealed no significant causal relationship between lipid and RA. We observed that the genetically predicted 3-hydroxy-3-methylglutaryl-coenzyme A reductase (HMGCR) -mediated low density lipoprotein cholesterol (LDL-C) (OR 0.704; 95% CI 0.56, 0.89; P = 3.43×10^−3^), Apolipoprotein C-III (APOC3) -mediated triglyceride (TG) (OR 0.844; 95% CI 0.77, 0.92; P = 1.50×10^−4^) and low density lipoprotein receptor (LDLR) -mediated LDL-C (OR 0.835; 95% CI 0.73, 0.95; P = 8.81×10^−3^) were significantly associated with a lowered risk of RA. while Apolipoprotein B-100 (APOB) -mediated LDL-C (OR 1.212; 95%CI 1.05,1.40; P = 9.66×10^−3^) was significantly associated with an increased risk of RA.

**Conclusions:**

Our study did not find any supporting evidence to suggest that lipids are a risk factor for RA. However, we observed significant associations between HMGCR, APOC3, LDLR, and APOB with the risk of RA.

## Introduction

RA is a chronic inflammatory disease that results in cartilage and bone tissue destruction, often leading to disability [1]. Its exact causes and pathogenesis have yet to be fully understood. Currently, glucocorticoids and non-steroidal anti-inflammatory drugs are utilized to alleviate clinical symptoms such as pain and inflammation in RA patients [2]. Disease-modifying rheumatic drugs (DMARDs) are considered the latest first-line treatment for RA [3]. Nonetheless, a proportion of patients have "difficult-to-treat RA", defined as continuing to experience symptoms of RA after failing two or more DMARDs treatments with different mechanisms [4]. Furthermore, low-income countries face challenges in accessing biological DMARDs [5]. These mean that a considerable number of individuals have unmet needs. Considering this context, it is essential to prioritize early identification of risk factors for RA and timely preventive measures.

The disrupted lipid profile in RA is well-established [6–8]. Previous observational studies have indicated that levels of LDL-C, high density lipoprotein cholesterol (HDL-C), and total cholesterol (TC) tend to decrease in the early years after an RA diagnosis and remain lower compared to healthy individuals [9]. This phenomenon can be attributed to the pro-inflammatory state experienced by RA patients [9]. The exact mechanism is unclear, but cytokine-induced activation of the reticuloendothelial system may be critical for this change [10]. Overproduction of acute-phase reactants under elevated inflammatory state load may have contributed to impaired cholesterol transport in the liver [11]. Interestingly, this finding contradicts the conventional understanding that higher lipid levels are associated with an elevated risk of developing coronary artery disease (CAD), giving rise to the term "lipid paradox" [12].

Studies have demonstrated that certain lipid-modifying drugs can effectively alleviate the clinical symptoms of RA. Among the commonly used lipid-modifying drugs are HMGCR inhibitors (statins), Ezetimibe, Proprotein convertase subtilisin/kexin type 9 (PCSK9) inhibitors (Alirocumab, Evolocumab), Volanesorsen, Evinacumab, Mipomersen, and Probucol. Statins, in particular, have shown significant anti-inflammatory effects in the treatment of RA [13]. The effectiveness of statins in alleviating the clinical symptoms of RA has garnered significant attention. However, there is ongoing debate regarding the potential association between statin use and the risk of developing RA. Multiple studies have suggested that long-term statin use might be linked to a reduced risk of RA [14–16]. One study found no increased risk of RA among statin users compared to non-users [17]. Conversely, another study demonstrated an increased risk of developing RA associated with statin use [18]. Ezetimibe has been found effective in reducing disease activity and indicators of inflammation in RA [19]. The pleiotropic effects of PCSK9, which are associated with inflammation and immunity, suggest that PCSK9 inhibitors could potentially serve as a therapy against inflammation and autoimmunity [20]. However, there is currently no evidence to support the therapeutic effects of several other lipid-modifying drugs on RA.

Previous research has demonstrated a potential association between lipids, lipid-modifying drugs, and RA. However, these studies are prone to biases caused by confounding factors and reverse causality. One approach to address these limitations is MR, which leverages the random assortment of alleles during meiosis, similar to the random assignment of subjects in a randomized controlled trial [21]. MR provides a viable method for evaluating the causal link between a drug treatment target and a disease. In drug target MR, genetic variations in the gene locus that encodes the drug protein are utilized as instrumental variables to model the regulatory effect of the drug on its target protein [22]. This approach allows us to gain insights into the potential role of lipid and lipid-modifying drug targets in the risk of RA. To investigate the potential relationship between lipids, lipid-modifying drugs, and RA, we conducted MR studies.

## Method

### Study design overview

**Fig 1:**
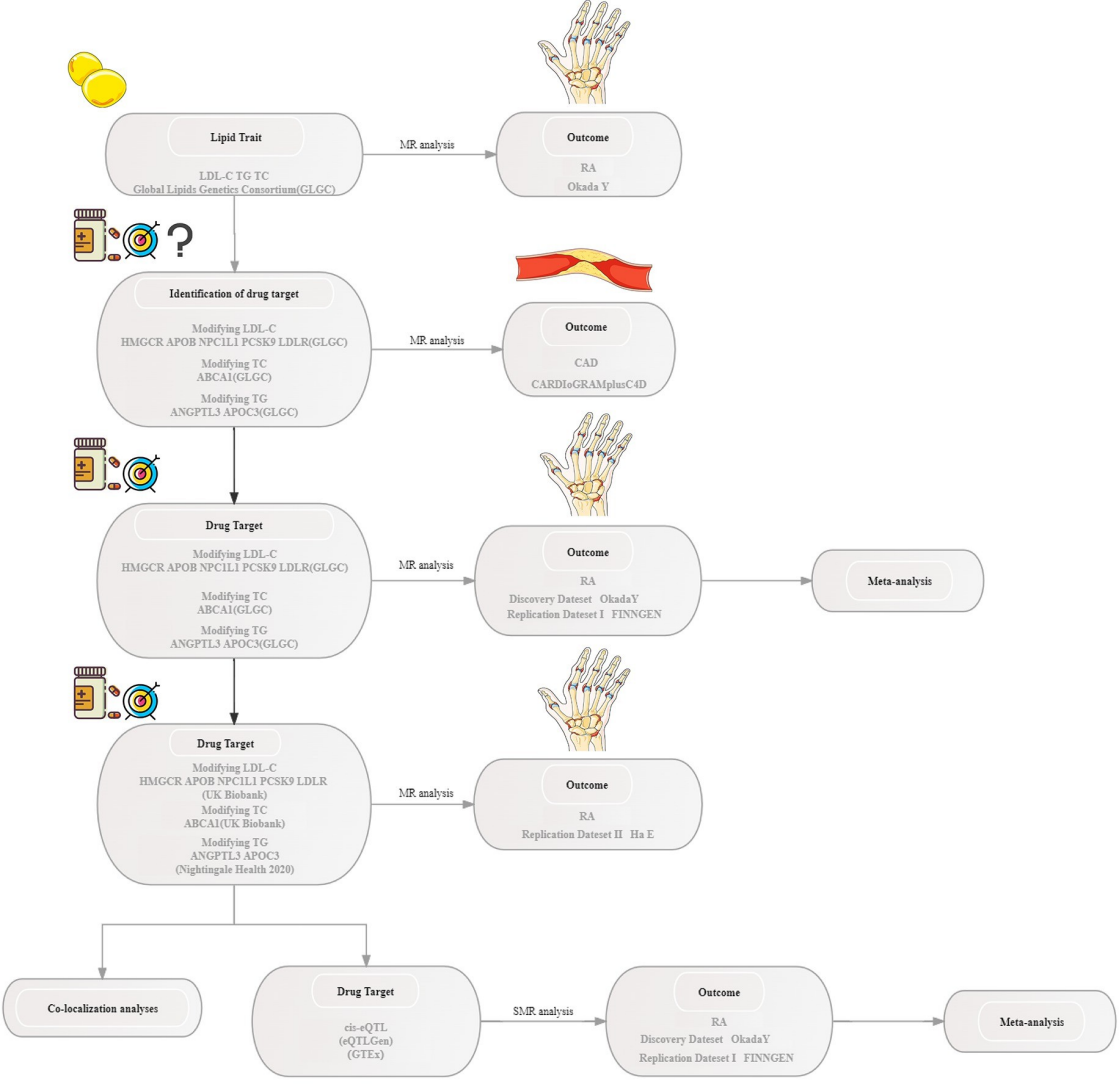
The summary of study design. NPC1L1, Niemann-Pick C1-like protein 1; ABCA1, ATP Binding Cassette Subfamily A Member 1; ANGPTL3, angiopoietin-like 3.

The present MR analysis aimed to elucidate the causal association between lipids, including cholesterol and triglycerides (TG), along with seven distinct types of lipid-modifying drug targets, and RA. Adhering to the Strengthening Observational Studies in Epidemiology—Guidelines for Reporting Mendelian Randomization (STROBE-MR) (S1 Table in S1 File) [23]. MR is based on three hypotheses: (Ⅰ) single nucleotide polymorphisms (SNPs) act as instrumental variables (IVs) and directly associate with exposure factors (Ⅱ) IVs are unassociated with confounding factors, (Ⅲ) IVs only affect the outcome through the exposure factors [24]. S2 Table in S1 File provides an extensive overview of targets of lipid-modifying drugs. A summary of the design of this study is shown in Fig 1.

### Data sources

The discovery datasets for LDL-C, TG, and TC were obtained from the GLGC [25]. The replication datasets for LDL-C and TG were obtained from the UK Biobank, and the replication datasets for TC were obtained from Nightingale Health in 2020. The summry genome-wide association studies (GWAS) for CAD were obtained from the Coronary ARtery DIsease Genome Wide Replication and Meta-analysis plus The Coronary Artery Disease (CARDIoGRAMplusC4D) [26]. The discovery dataset for RA was from a study by Okada Y [27]. The replication dataset I for RA was from the R8 of the FINNGEN [28], and replication dataset II for RA was from a GWAS meta-analysis by Ha E [29]. All cis-eQTL used in this study were obtained from Gtex and eQTLgen [30]. For more information, please refer to supporting information (S3 Table in S1 File).

### Genetic instruments selection

As part of the instrumental variable selection process for lipid traits, we chose SNPs that showed significant associations with lipid traits at the genome-wide level (P<5×10^−8^). The threshold for selecting these SNPs was a linkage disequilibrium (LD) parameter of 0.001 within a range of 10,000 kb. We identified common lipid-modifying medications based on guidelines for dyslipidemia management, which included HMGCR inhibitors (statins), Ezetimibe, PCSK9 inhibitors (Alirocumab, Evolocumab), Volanesorsen, Evinacumab, Mipomersen, and Probucol. Through Drugbank (https://go.drugbank.com/), we queried the respective target genes associated with each drug, classifying them as LDL-C lowering (HMGCR, APOB, NPC1L1, PCSK9, LDLR), TG lowering (ANGPTL3, APOC3), and TC lowering (ABCA1). We selected SNPs that showed significant associations (P<5×10^−8^) with LDL-C, TG, and TC at the genome-wide level as instrumental variables for the corresponding lipid-modifying drugs. We set the LD parameter at r2<0.3 within a range of 100 kb. For SNPs missing in the outcome GWAS, we used proxy SNPs (r2 > 0.8) as substitutes. To ensure the reliability of analysis results, we excluded SNPs with palindromic structures [31]. To obtain instrumental variables, we selected SNPs with minor allele frequencies > 0.01 that displayed significant associations (P<5×10^−8^) with exposure in cis-eQTL. The F-statistic was calculated using the equation [32]:

$$F=\frac{beta^{2}}{se^{2}}$$

and only instrumental variables with F>10 were retained to avoid weak instrumental bias [33]. Detailed information on all included SNPs can be found in supporting information (S4-S14 Tables in S1 File).

### Instrumental variable validation

To validate the rationale behind the selection of genetic variants as drug targets, a positive control MR analysis was conducted [34]. Considering that lipid-modifying drugs primarily target CAD, we utilized pooled data for CAD obtained from CARDIoGRAMplusC4D, as the outcome for the positive control analysis.

### MR analysis

**Fig 2:**
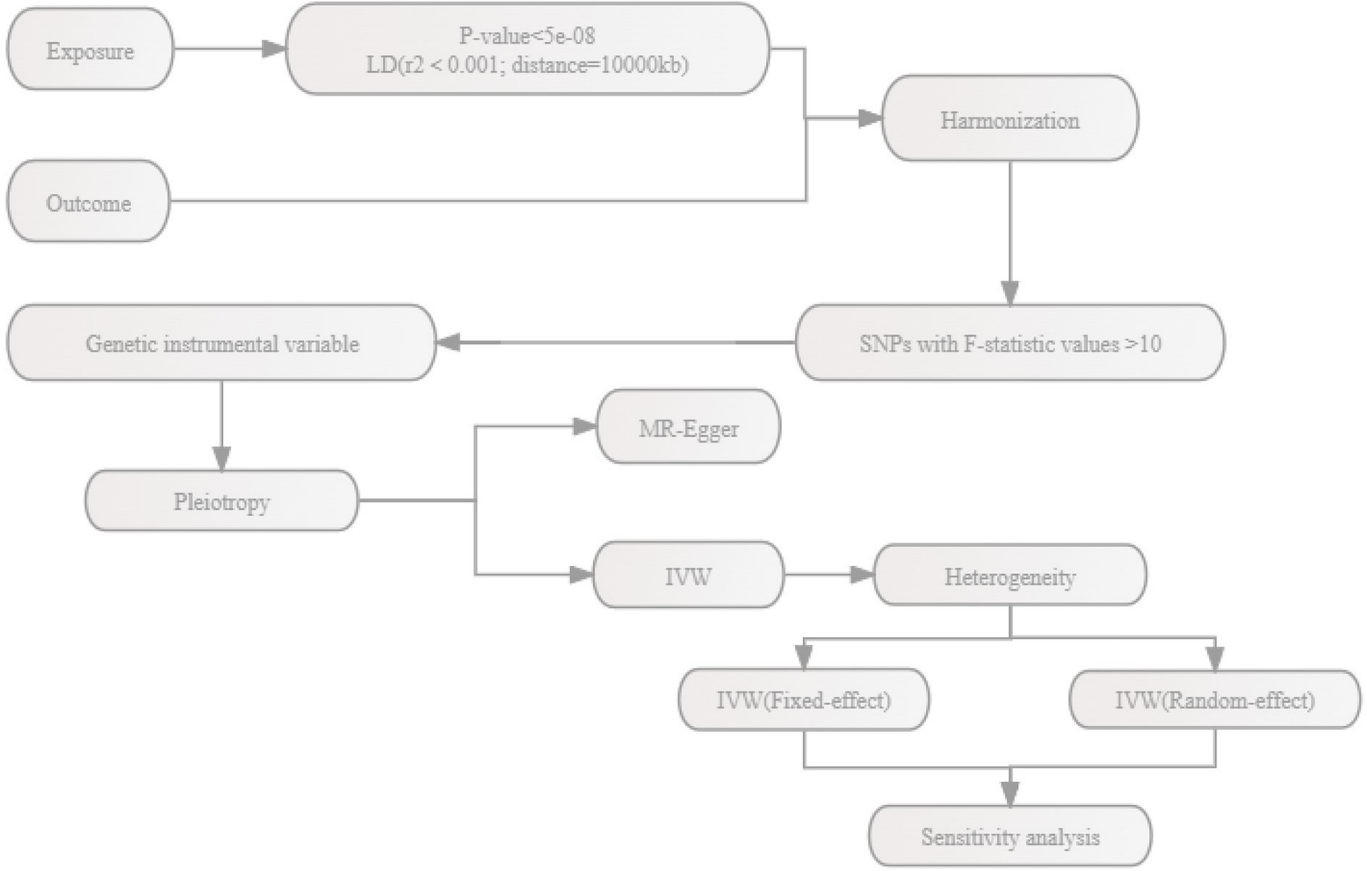
Flowchart of MR analysis.

We initially conducted a pleiotropy analysis to assess the impact of genetic variables related to drug targets on the risk of RA. To account for pleiotropy, we employed MR-Egger when it was present, and the inverse variance weighted (IVW) method when pleiotropy was absent. Heterogeneity testing was employed to determine the appropriate choice between a random-effects model or a fixed-effects model for IVW. Specifically, a random-effects model was selected when heterogeneity was observed (Q_pval<0.05 and I2>50%), and conversely, a fixed-effects model was utilized. We performed leave-one-out analysis to evaluate the influence of individual SNPs on the results. The process of MR analysis is illustrated in Fig 2. Statistical power for MR analysis was calculated using an online tool (https://shiny.cnsgenomics.com/mRnd/) (S15 Table in S1 File). Meta-analysis, employing fixed or random effects models, was conducted to combine estimates of the association between outcomes from various sources. If the estimates from the preliminary MR analysis were significant when combined, data from different sources were employed for validation. The odds ratios (OR) for RA risk were calculated with respect to a standard deviation (SD) decrease in LDL-C, TG, and TC. We considered correlations with a significance level of P<0.05/5 (HMGCR, APOB, NPC1L1, PCSK9, LDLR), P<0.05 (ABCA1), and P<0.05/2 (ANGPTL3, APOC3) as significant after applying Bonferroni correction. The "TwosampleMR" and "Metafor" packages in R (version 4.2.2) were used for the MR analysis.

### SMR analyses

We conducted an SMR analysis to re-evaluate the association that remained significant following thorough validation. This analysis used eQTL data as the exposure and GWAS data as the outcome. Additionally, we employed the heterogeneity in dependent instruments test to examine potential pleiotropy (P<0.05) [35]. The SNPs utilized in the SMR analysis can be found in supporting information (S16-S18 Tables in S1 File). A correlation was deemed significant if it had a P-value<0.05 after combining estimates from different sources of outcomes through meta-analysis. SD changes in gene expression levels for each additional effect allele. The eQTL data were quantified by measuring the changes in expression levels associated with each additional effect allele, equivalent to a 1-SD. The entire analysis was executed using SMR software (version 1.3.1).

### Colocalisation analysis

To validate the causal relationship between drug-targeted genes and RA, we employed Bayesian co-localization analysis, complementing the findings from the SMR analysis. The co-localization analysis involved consideration of five hypotheses: (H0) SNPs within the selected location are unrelated to both trait 1 and trait 2; (H1) there are SNPs within the selected location that are associated with trait 1 but unrelated to trait 2; (H2) there are SNPs within the selected location that are associated with trait 2 but unrelated to trait 1; (H3) the two traits are related but have distinct causal variants; (H4) the two traits are related and share a common causal variant [36].This analysis was conducted within a ±100 kb window surrounding the drug target gene. A posterior probability greater than 0.80, indicating two traits sharing the same causal variant, was considered as evidence of co-location.

The primary focus of interest is the probability of co-localization, which refers to the probability that both the exposure and outcome features are influenced by the same causal variant, assuming that there is a causal variant in the outcome. This probability is computed as H4 divided by the sum of H3 and H4 (H4 / (H3 + H4)). It serves as an indicator of the extent to which a genetic variant affects both the exposure and outcome characteristics.

The co-localization analysis was performed using the coloc package in R (version 4.2.2).

### Ethics

Ethical approval was not necessary for this study since all data sources utilized publicly available summary-level data. Moreover, each of the included studies obtained approval from the respective institutional review boards.

## Results

### MR analysis of lipids and RA

Heterogeneity was observed in the MR analyses of LDL-C, TG, and TC in comparison to RA, although no evidence of pleiotropy was detected (S19 Table in S1 File). Therefore, the analyses were conducted using the random effects model of IVW. The MR analysis did not reveal any significant causal association between LDL-C, TG, TC, and RA (S20 Table in S1 File).

### Positive control analysis

All the drug target genes investigated in this study exhibited a significant association with CAD risk (S21 Table in S1 File), which demonstrates the rationale for the selection of instrumental variables for lipid-modifying drugs. Given the presence of pleiotropy specifically in the APOB analysis of CAD (S22 Table in S1 File), we employed the MR-Egger method for this particular analysis. MR-Egger is a suitable approach for assessing causality in the presence of pleiotropy [24]. For all other analyses, we utilized the IVW method.

### MR analysis of drug targets-mediated lipid

**Fig 3:**
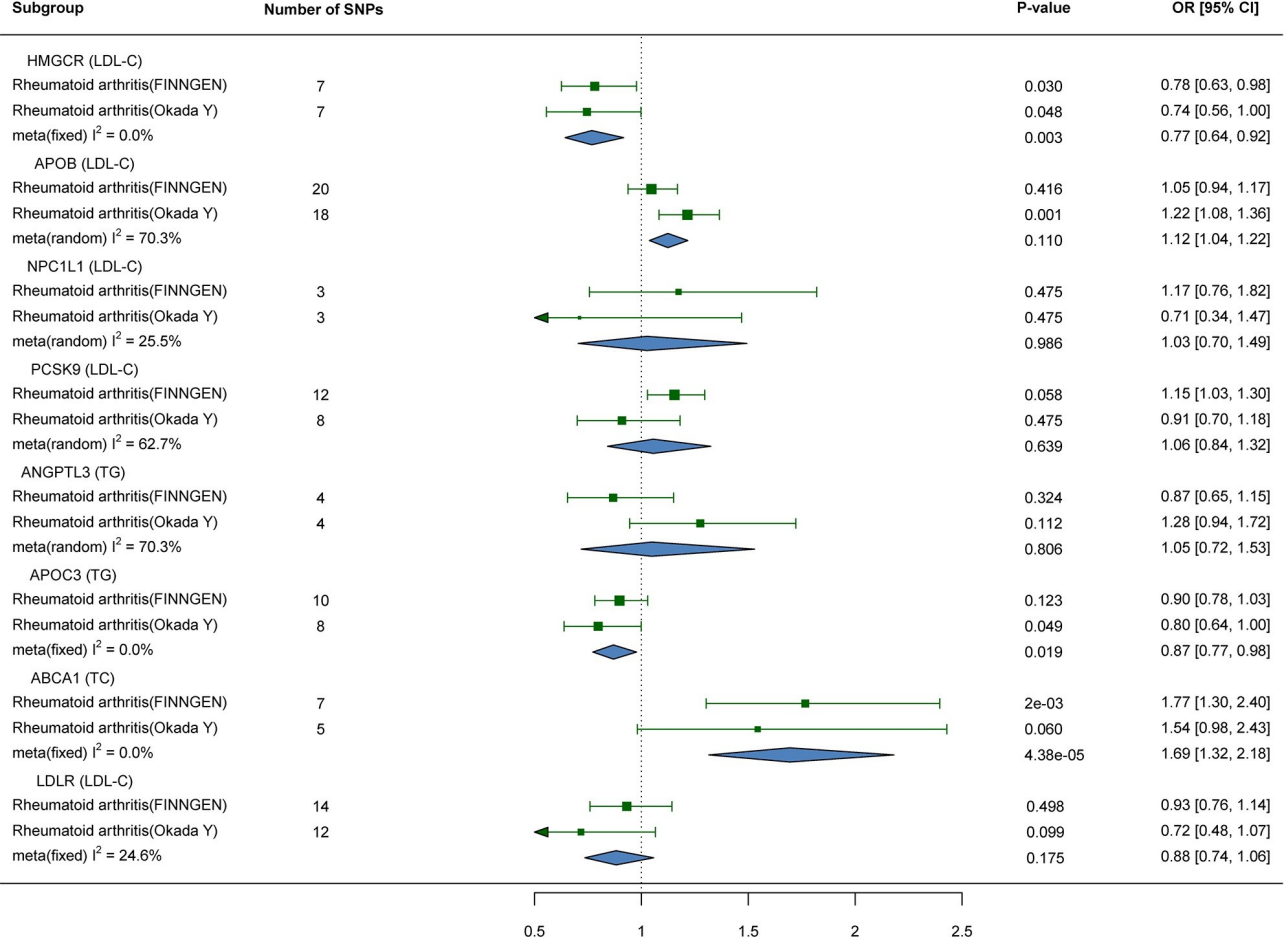
Results of MR analysis of drug targets-mediated lipid and RA.

**Fig 4:**
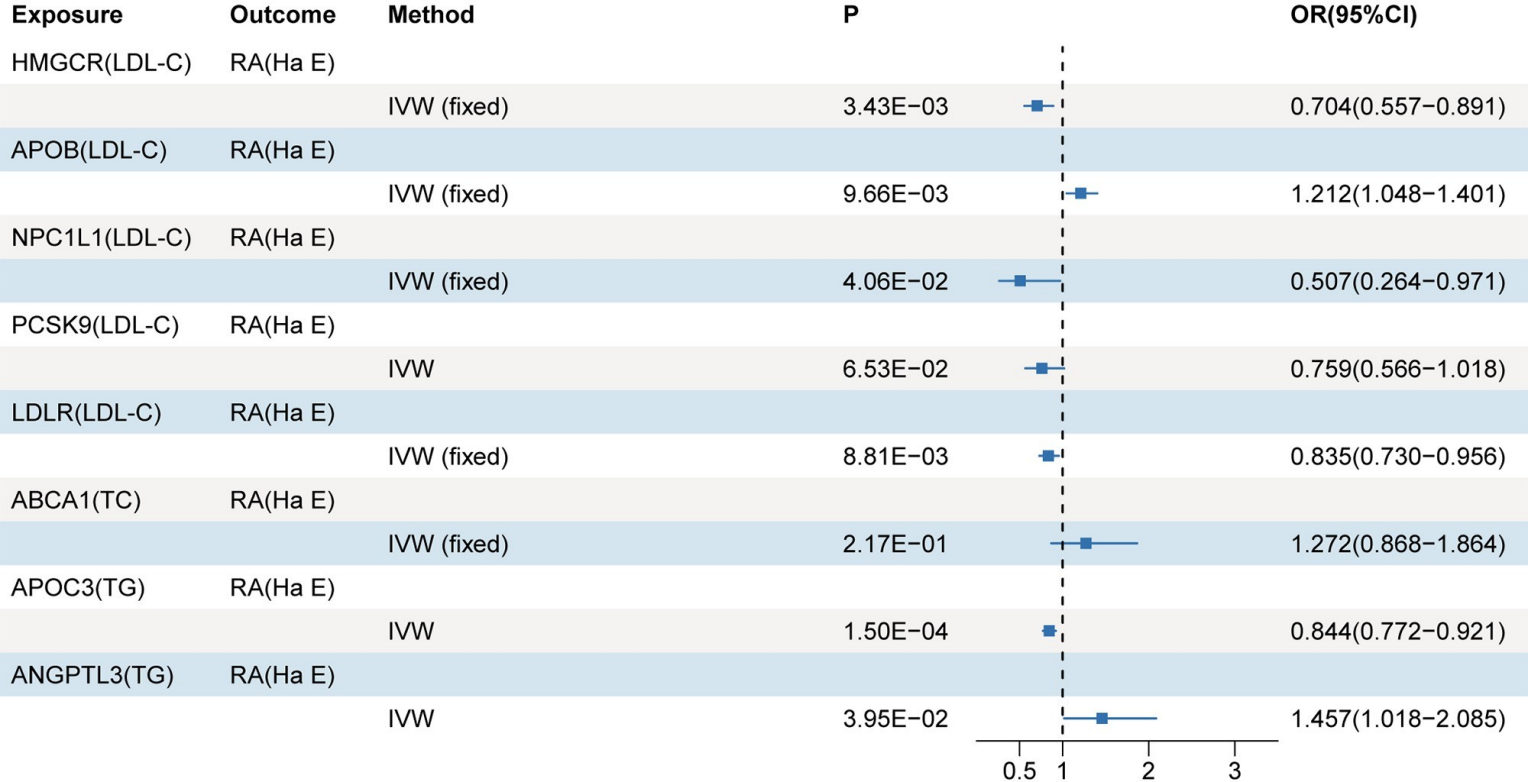
Results of MR analysis of drug targets-mediated lipid and RA (Ha E).

In the MR analyses conducted between the drug targets and the discovery dataset, replication dataset I, and replication dataset Ⅱ of RA, no evidence of pleiotropy or heterogeneity was found (S23-S25 Tables in S1 File). All drug targets-mediated lipid MR analyses were performed using the IVW method, specifically implementing the fixed-effects model. After conducting a meta-analysis to combine all MR results, significant associations were observed between genetic variants in the HMGCR-mediated LDL-C (OR 0.77; 95%CI 0.64,0.92; P = 0.004), ABCA1-mediated TC (OR 1.69; 95%CI 1.32,2.18; P = 4×10^−4^), and APOC3-mediated TG (OR 0.87; 95%CI 0.77,0.98; P = 0.019) targets and the risk of RA (Fig 3). However, when analyzing the Okada Y dataset, genetic variation in APOB-mediated LDL-C was significantly associated with RA risk (OR 1.22; 95%CI 1.08,1.36; P = 0.001), whereas this association was not observed in the Finngen dataset (OR 1.05; 95%CI 0.94,1.17; P = 0.416) (Fig 3). The leave-one-out analysis demonstrates that the results are not influenced by any individual SNP (S1–S3 Figs). Meta-analysis of these two results resulted in a non-significant association between genetic variation in APOB-mediated LDL-C and RA (OR 1.13; 95%CI 0.97,1.3; P = 0.11) (Fig 3). No evidence was found to support the association between risk of RA and other genetic variants targeted by drugs (Fig 3). After replacing the exposure and outcome datasets, the causal association between genetic variation in the HMGCR-mediated LDL-C (OR 0.704; 95%CI 0.56,0.89; P = 0.003) and APOC3-mediated TG (OR 0.844; 95%CI 0.77,0.92; P = 1×10^−3^) with the risk of RA remained significant (Fig 4). LDLR-mediated LDL-C (OR 0.835; 95%CI 0.73,0.96; P = 8.8×10^−3^) and APOB-mediated LDL-C (OR 1.21; 95%CI 1.04,1.40; P = 9.6×10^−3^) demonstrated significant associations with the risk of RA (Fig 4). Additionally, NPC1L1-mediated LDL-C and ANGPTL3-mediated TG exhibited suggestive causal associations with the risk of RA (Fig 4). However, the previously observed significant causal association between genetic variation in the ABCA1-mediated TC and the risk of RA disappeared (Fig 4). The leave-one-out analysis demonstrates the robustness of the results (S4 Fig). For more details on the MR analysis, please refer to supporting information (S26-S28 Tables in S1 File).

### MR analysis of drug targets-mediated lipid and risk factors for RA

**Fig 5:**
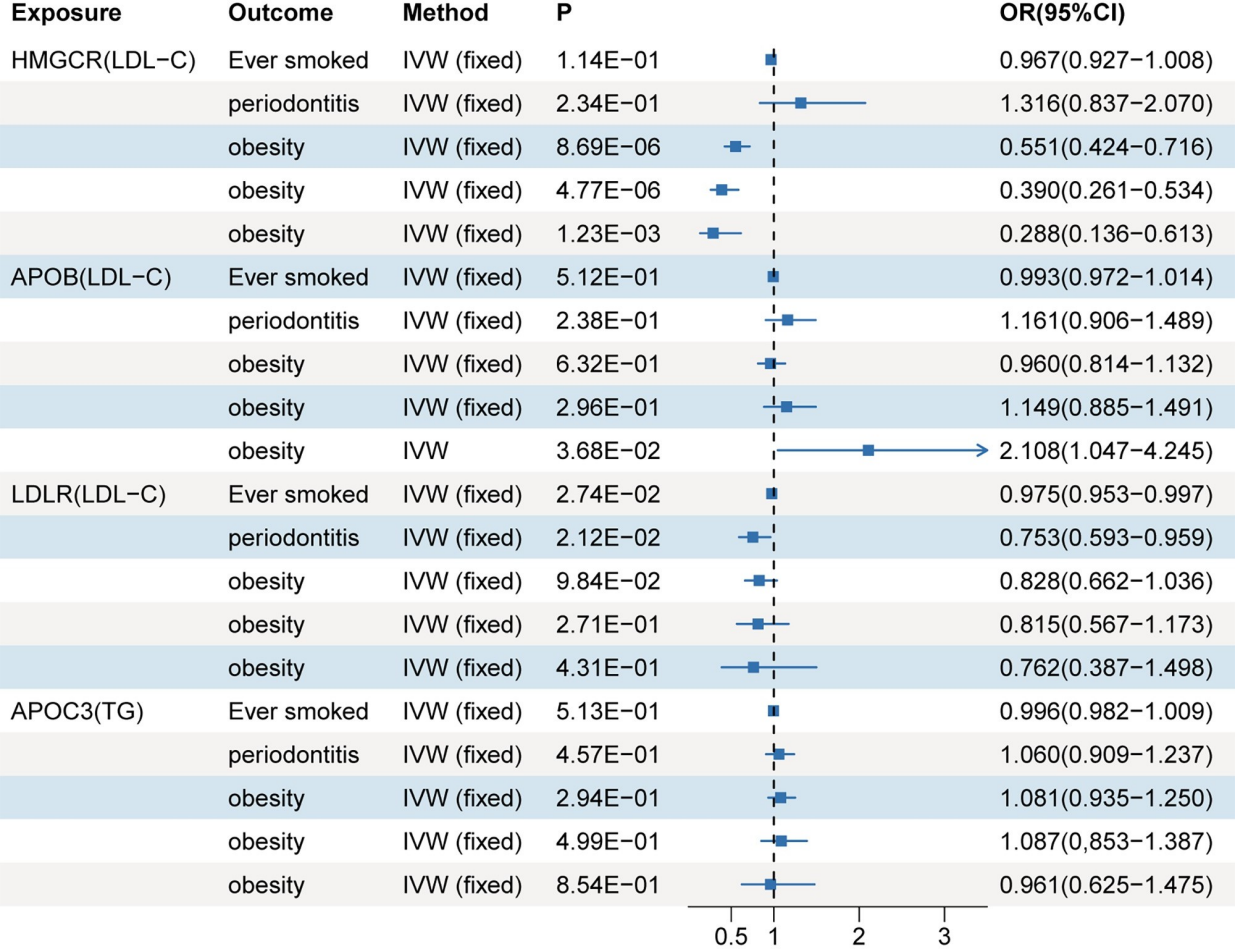
Results of MR analysis of drug targets-mediated lipid and risk factors of RA.

Considering that smoking, periodontitis, and obesity are well-known risk factors for RA, we employed IVW approach to examine the association between lipid-modifying drug targets and these RA risk factors. Fig 5 illustrates these associations. Our analysis revealed a significant association between HMGCR-mediated LDL-C and the risk of obesity at levels 1 (OR 0.551; 95%CI 0.424, 0.716; P = 8.96×10^−6^), level 2 (OR 0.390; 95%CI 0.261, 0.534; P = 4.77×10^−6^), and level 3 (OR 0.288; 95%CI 0.136, 0.613; P = 1.23×10^−3^). Additionally, APOB-mediated LDL-C showed a suggestive association only with the risk of obesity at level 3 (OR 2.108; 95%CI 1.047, 4.245; P = 3.68×10^−2^), while LDLR-mediated LDL-C exhibited a suggestive association solely with ever smoking (OR 0.975; 95%CI 0.953, 0.997; P = 2.74×10^−2^) and periodontitis (OR 0.753; 95%CI 0.593, 0.959; P = 2.12×10^−2^).

### SMR analyses

**Fig 6:**
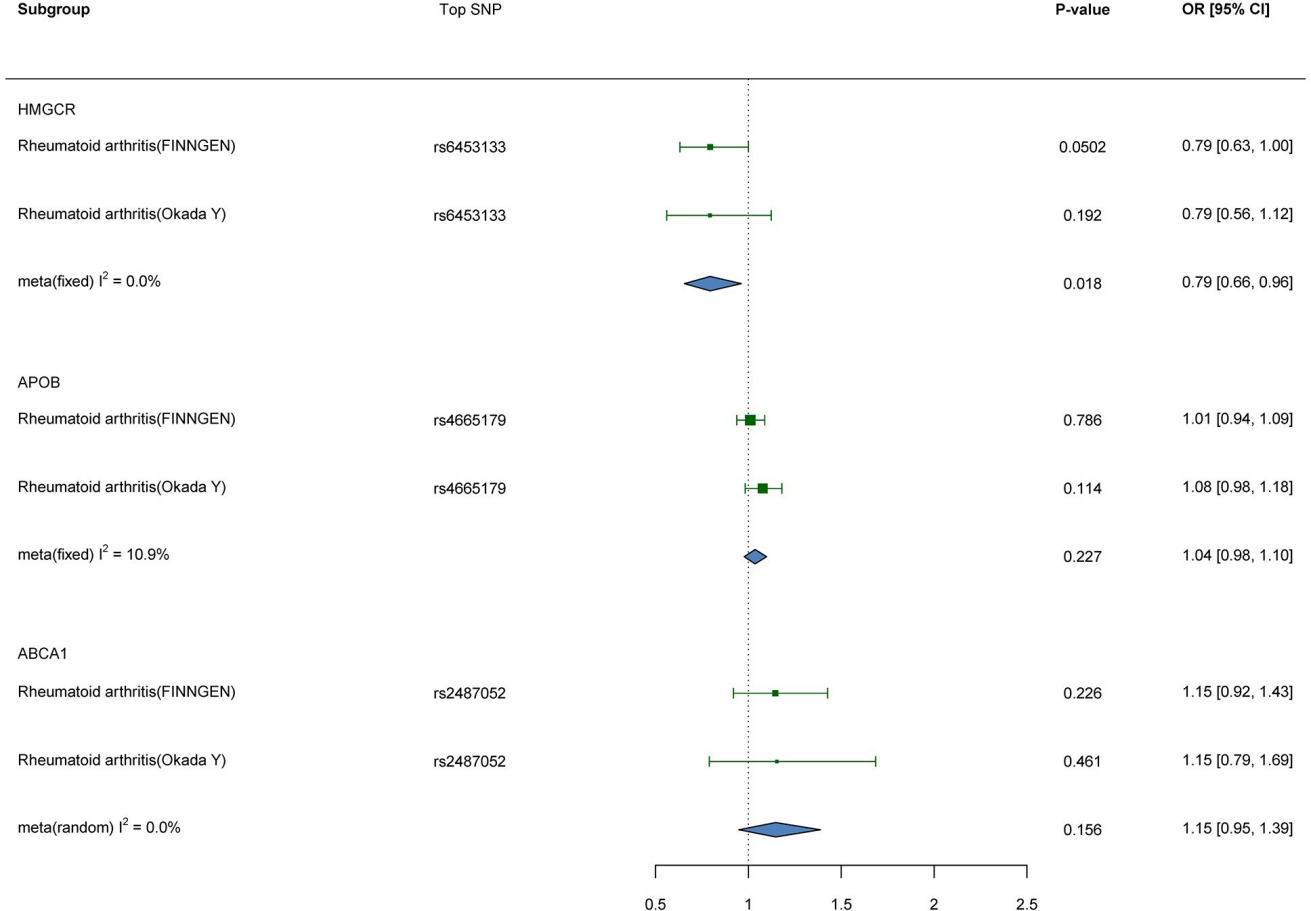
Results of SMR analysis of drug targets and RA.

We conducted SMR analyses using cis-eQTLs of HMGCR in blood tissues from the eQTLGen database, with GWAS data for RA from both the Okada Y and FINNGEN databases. The SMR results were then combined using a fixed-effects model meta-analysis. Consistent with previous MR analyses, our findings indicate a significant association between genetically predicted blood HMGCR and a lower risk of RA (OR 0.79; 95%CI 0.66, 0.96; P = 0.019). In addition, we observed that ABCA1 in blood and APOB in adipose tissue were associated with an increased risk of RA, but this association did not reach statistical significance (Fig 6). None of the SMR analyses showed evidence of significant pleiotropy (S29-S31 Tables in S1 File). However, we were unable to find eligible cis-eQTLs for APOC3 and LDLR in blood tissue, adipose tissue, and liver tissue.

### Colocalisation analysis

In the co-localization analysis, we found a 99.9% probability (S32 Table in S1 File) that the top SNP:rs6453133 of HMGCR, used for the SMR analysis, shared the same causal variant with RA. For LDL-C and RA within the HMGCR gene, the probability of co-localization was 91.3%, for LDL-C and RA within the APOB gene, 82.5%, for LDL-C and RA within the LDLR gene, 84.8%, and for TG and RA within the APOC3 gene, 77.8%. These findings suggest that the effects of HMGCR, APOC3, LDLR and APOB on RA are not affected by the confounding of LD variants. More details of the co-localization analysis can be found in S33 Table in S1 File and S5 Fig.

## Discussion

Through MR analysis, we did not identify a causal association between lipid and RA. However, we found that HMGCR, APOC3 and LDLR were significantly associated with lower RA risk. APOB is significantly associated with higher RA risk.

Statins, widely used worldwide, are fundamental drugs utilized to inhibit cholesterol synthesis and stabilize atherosclerosis. Through inhibition of HMGCR, statins reduce endogenous cholesterol synthesis and simultaneously increase expression of hepatic LDLR [37]. Consequently, there is an increase in hepatic clearance of lipoproteins, leading to lipid-modifying effects. Current rheumatology guidelines recommend combining statin treatment with antirheumatic drugs as part of routine RA treatment [38]. Undeniably, the potential benefits of statins for patients with RA have been suggested previously [13, 39]. The potential negative impact of statins on RA may be attributed to their effect on insulin resistance. The impairment of HMGCR activity, induced by statins, can have adverse effects on insulin sensitivity and increase insulin resistance [40–43]. Treatment with insulin triggers a conversion of macrophages from an M1 to an M2 polarization state [44], characterized by high expression of anti-inflammatory cytokines that inhibit T-cell proliferation and activation, regulate the Th2-type immune response, and exert anti-inflammatory effects [45, 46]. In a comprehensive meta-analysis incorporating two HMGCR SNPs and 43 studies, it was proposed that HMGCR inhibitors lead to increased body mass index (BMI), which might mediate the relationship between HMGCR inhibitors and insulin resistance [40]. Furthermore, an MR study revealed a significant association between BMI and a higher risk of RA [47]. Our study identified an association between HMGCR and a decreased risk of RA, suggesting that HMGCR inhibitors may increase the risk of RA. However, further investigations are necessary to explore the underlying molecular mechanisms, which could enhance the management of cardiovascular disease prevention in individuals susceptible to RA. Notably, several studies have demonstrated the cardiovascular benefits of HMGCR inhibitors in RA patients [48, 49], as their use has been associated with lower mortality in RA patients [48]. This study offers insights into the mechanisms involved and does not advocate for any modifications to the current guidelines on statin prescription for preventing cardiovascular disease in patients with RA. Additionally, our study suggests the potential of APOC3 inhibitors to increase the likelihood of RA, although the exact mechanism behind this association remains unclear. Previous research has shown an independent association between APOC3, insulin resistance, and β-cell dysfunction in RA patients [50].

Studies suggest that LDLR is the primary receptor for PCSK9. Inhibitors of PCSK9 enhance the expression of LDLR in the liver by preventing the binding of PCSK9 to LDLR, resulting in a reduction of LDL-C levels. A recent study found that levels of PCSK9 were elevated in patients with RA and were positively correlated with levels of C-reactive protein as well as disease activity [51]. Another in vitro study demonstrated that PCSK9 promotes the differentiation of th17 cells through a pathway associated with LDLR, while PCSK9 inhibitors reduced the differentiation of th17 cells [52]. Additionally, the deficiency of LDLR disrupted the differentiation of osteoblasts in vitro, possibly due to a reduction in the expression of runt-related transcription factor 2 [53]. Several members of the LDLR family have been shown to be involved in the regulation of osteoblastogenesis [54–56]. Among them, SNPs rs3736228 and rs4988321 of low-density lipoprotein receptor-related protein-5 were significantly associated with susceptibility to RA [57].Patients with RA exhibit substantially elevated levels of APOB [58], which aggravates arthritis by interacting with enolase-1 expressed on the surface of immune cells, consequently amplifying the inflammatory response [59]. APOB inhibitors may influence the risk of RA through this mechanism. While these mechanisms offer potential pathways through which LDLR enhancement and APOB inhibition may reduce the risk of RA, there is a need for further exploration and validation of their precise roles and interactions.

It is important to acknowledge the limitations that need to be considered when interpreting the findings of our study. Firstly, it should be noted that the long-term impact of alterations in lipid levels resulting from the genetic variants used in the MR analysis may not directly reflect the effects of lipid-modifying drugs on RA. This discrepancy arises from the fact that medications are regularly taken, whereas genetic variants are determined early in life, even before conception. Secondly, despite conducting rigorous sensitivity analyses, there is still the possibility of horizontal pleiotropy and the bias it introduces. It is not possible to entirely rule out these issues. Thirdly, our analysis was limited to summary GWAS data from European populations, so the effects of changes in lipid levels and HMGCR may differ in other populations. Therefore, future investigations with larger sample sizes across diverse populations are warranted. Fourthly, regarding the SMR analysis, several eQTL drug targets showed associations with RA; however, some of these associations did not attain statistical significance, potentially attributed to the limited statistical power and the small sample size of the eQTL data. Fifthly, the low posterior probability of sharing causal variants in co-localization could be influenced by factors such as limited statistical power and the absence of causal variants in the Exposure and Outcome GWAS dataset. Furthermore, in our study, we encountered a challenge in finding cis-eQTL for APOC3 and LDLR in human tissue for the SMR analysis, which serves to validate the robustness of the results obtained through MR. To ensure more comprehensive conclusions, it is necessary to conduct future analyses using cis-eQTL in different human tissues.

## Conclusions

Our study did not provide evidence supporting lipid levels as a risk factor for RA. However, we identified associations between HMGCR, APOC3, and LDLR and a decreased risk of RA, while APOB showed an association with an increased risk of RA. These findings suggest that HMGCR, APOC3, LDLR, and APOB may serve as promising targets for the treatment of RA. However, further studies are necessary to gain a deeper understanding of the underlying mechanisms.

## Supporting information

S1 File Supplementary tables. (XLSX)

S1 Fig Leave-one-out plots in preliminary analysis of HMGCR and APOB-mediated lipid. A. Leave-one-out plots of HMGCR-mediated LDL-C on RA (Okada Y); B. Leave-one-out plots of HMGCR-mediated LDL-C on RA (FINNGEN); C. Leave-one-out plots of APOB-mediated LDL-C on RA (Okada Y); D. Leave-one-out plots of APOB-mediated LDL-C on RA (FINNGEN).
(TIF)

S2 Fig Leave-one-out plots in preliminary analysis of LDLR and APOC3-mediated lipid. A. Leave-one-out plots of LDLR-mediated LDL-C on RA (Okada Y); B. Leave-one-out plots of LDLR-mediated LDL-C on RA (FINNGEN); C. Leave-one-out plots of APOC3-mediated TG on RA (Okada Y); D. Leave-one-out plots of APOC3-mediated TG on RA (FINNGEN).
(TIF)

S3 Fig Leave-one-out plots in preliminary analysis of ABCA1-mediated TC. A. Leave-one-out plots of ABCA1-mediated TC on RA (Okada Y); B. Leave-one-out plots of ABCA1-mediated TC on RA (FINNGEN).
(TIF)

S4 Fig Leave-one-out plots in validation analysis. A. Leave-one-out plots of HMGCR-mediated LDL-C on RA (Ha E); B. Leave-one-out plots of APOB-mediated LDL-C on RA (Ha E); C. Leave-one-out plots of LDLR-mediated LDL-C on RA (Ha E); D. Leave-one-out plots of APOC3-mediated TG on RA (Ha E).
(TIF)

S5 Fig Results of co-localization analysis. A. Results of co-localization analysis of HMGCR-mediated LDL-C and RA; B. Results of co-localization analysis of APOB-mediated LDL-C and RA; C. Results of co-localization analysis of LDLR-mediated LDL-C and RA; D. Results of co-localization analysis of APOC3-mediated TG and RA.
(TIF)
