# Supplementary figures and images for: Potential association of genetically predicted lipid and lipid-modifying drugs with rheumatoid arthritis: A Mendelian randomization study
Source: PLoS One. 2024 Feb 28;19(2):e0298629. doi: 10.1371/journal.pone.0298629 (PMC10901327; doi:10.1371/journal.pone.0298629)

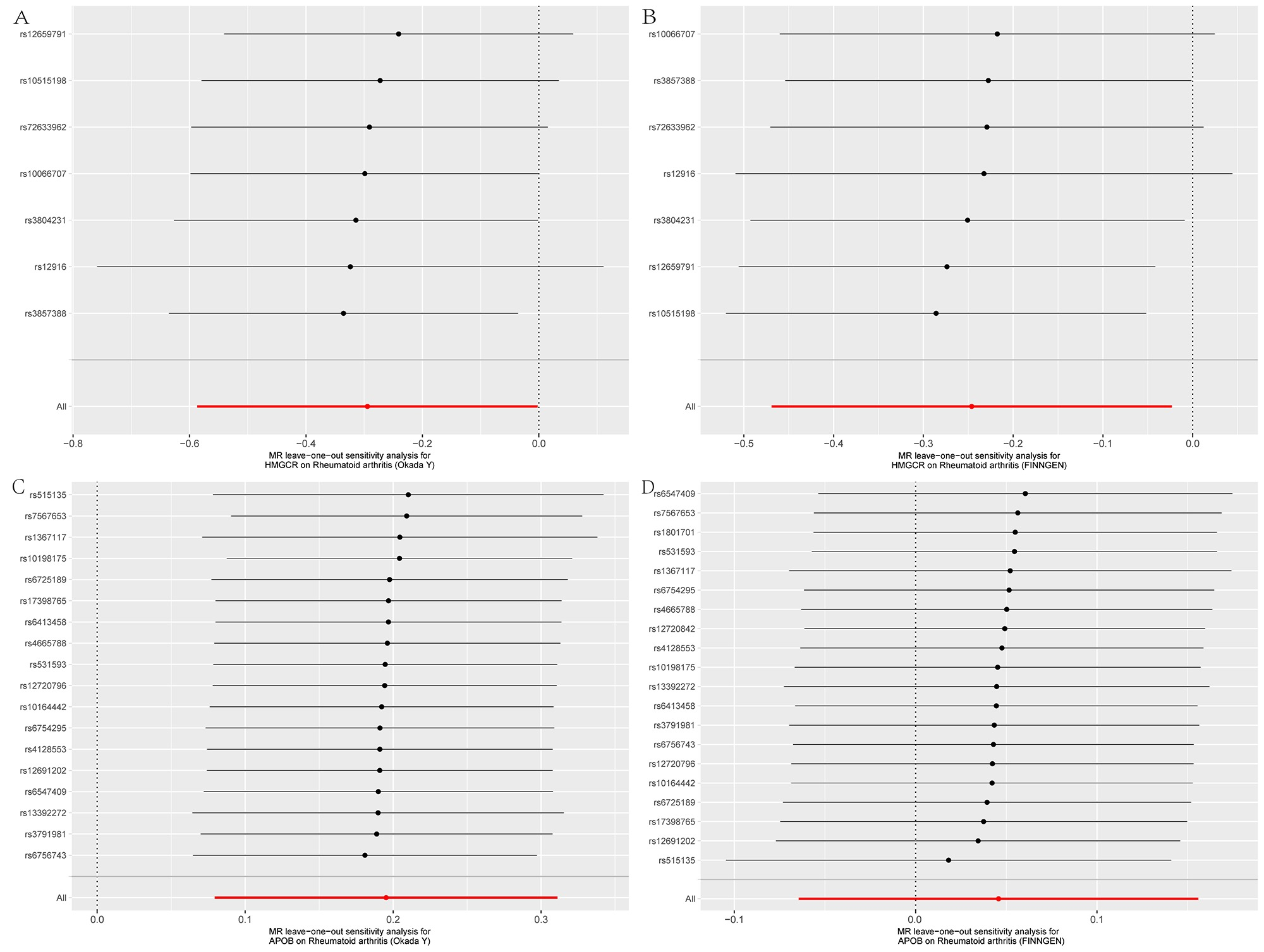

Supplement: S1 Fig — A. Leave-one-out plots of HMGCR-mediated LDL-C on RA (Okada Y); B. Leave-one-out plots of HMGCR-mediated LDL-C on RA (FINNGEN); C. Leave-one-out plots of APOB-mediated LDL-C on RA (Okada Y); D. Leave-one-out plots of APOB-mediated LDL-C on RA (FINNGEN). (TIF) [file pone.0298629.s002.tif]

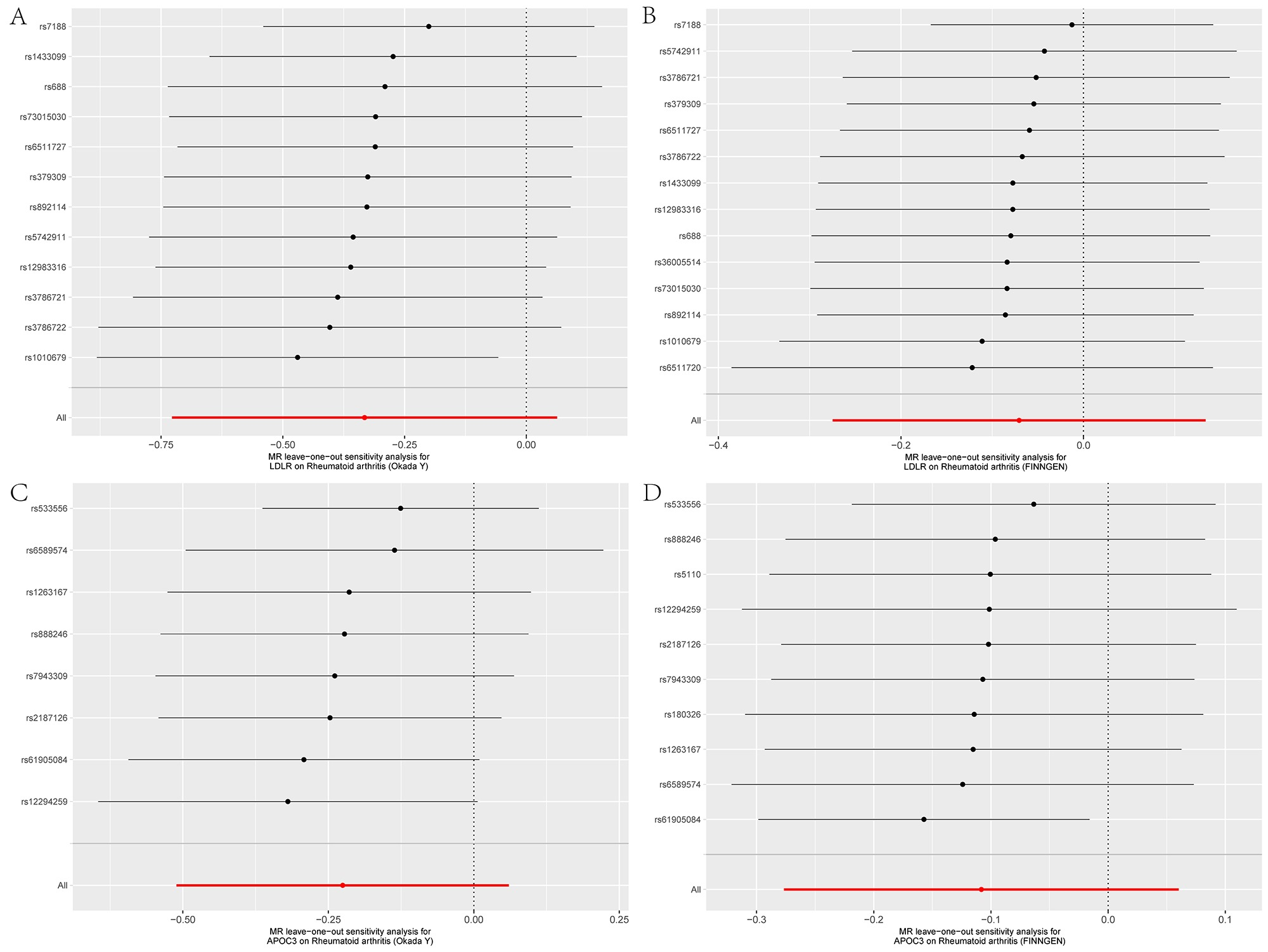

Supplement: S2 Fig — A. Leave-one-out plots of LDLR-mediated LDL-C on RA (Okada Y); B. Leave-one-out plots of LDLR-mediated LDL-C on RA (FINNGEN); C. Leave-one-out plots of APOC3-mediated TG on RA (Okada Y); D. Leave-one-out plots of APOC3-mediated TG on RA (FINNGEN). (TIF) [file pone.0298629.s003.tif]

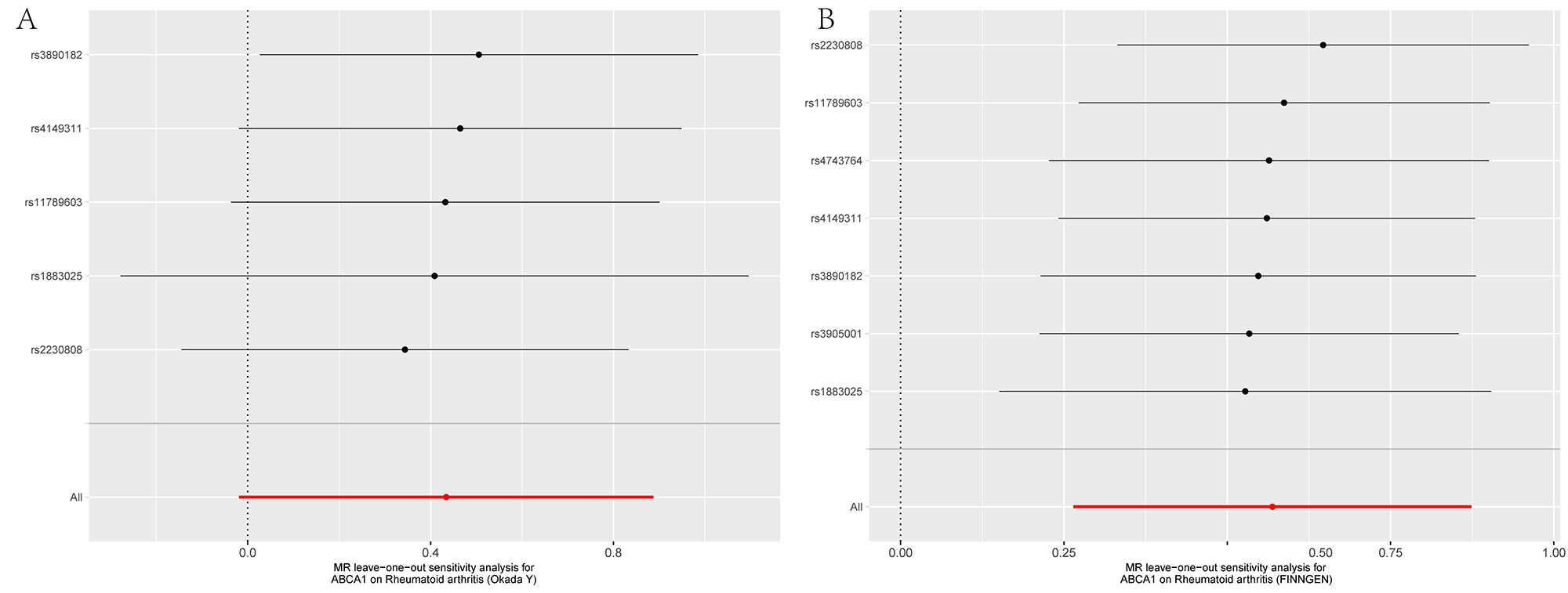

Supplement: S3 Fig — A. Leave-one-out plots of ABCA1-mediated TC on RA (Okada Y); B. Leave-one-out plots of ABCA1-mediated TC on RA (FINNGEN). (TIF) [file pone.0298629.s004.tif]

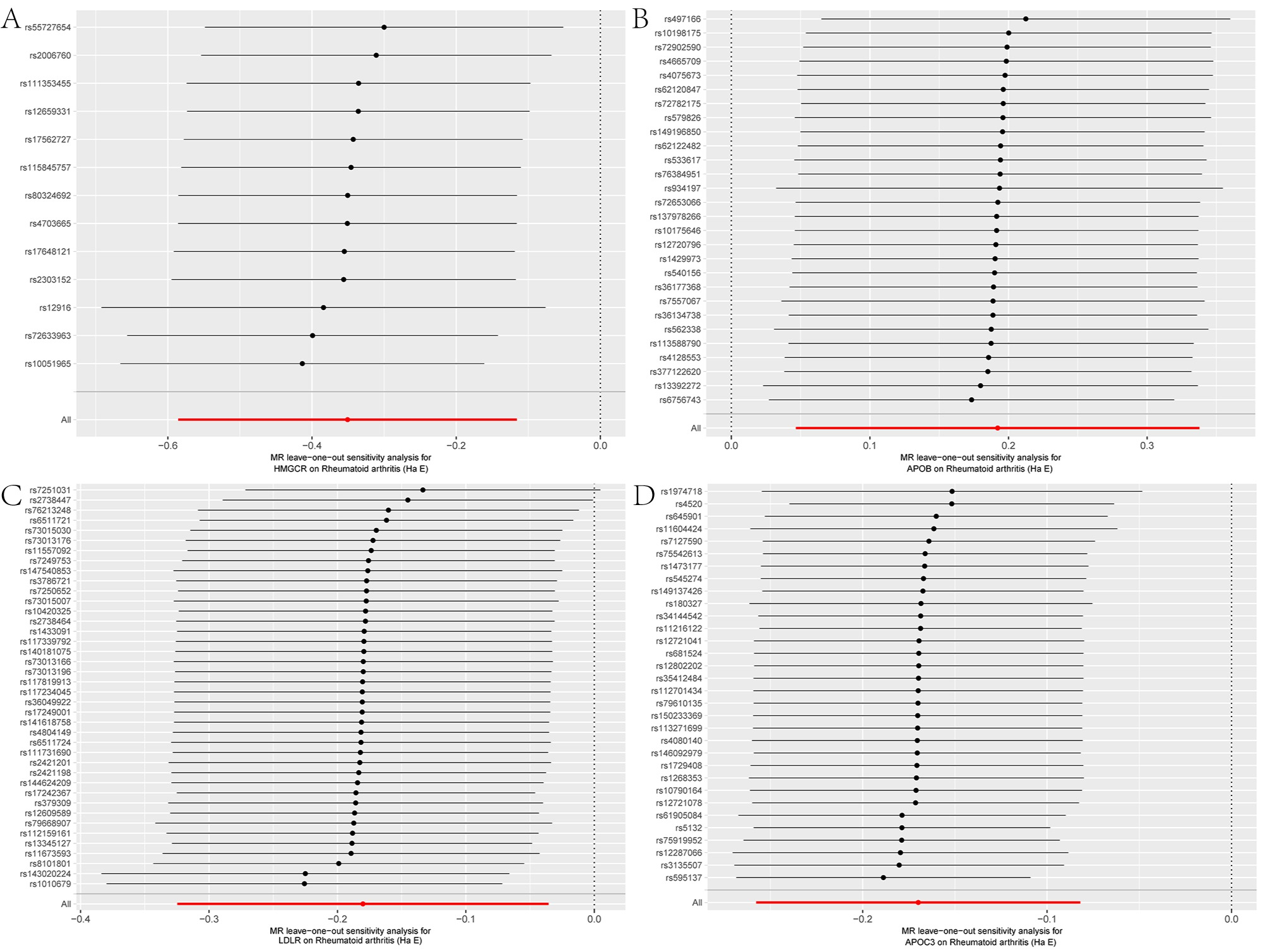

Supplement: S4 Fig — A. Leave-one-out plots of HMGCR-mediated LDL-C on RA (Ha E); B. Leave-one-out plots of APOB-mediated LDL-C on RA (Ha E); C. Leave-one-out plots of LDLR-mediated LDL-C on RA (Ha E); D. Leave-one-out plots of APOC3-mediated TG on RA (Ha E). (TIF) [file pone.0298629.s005.tif]

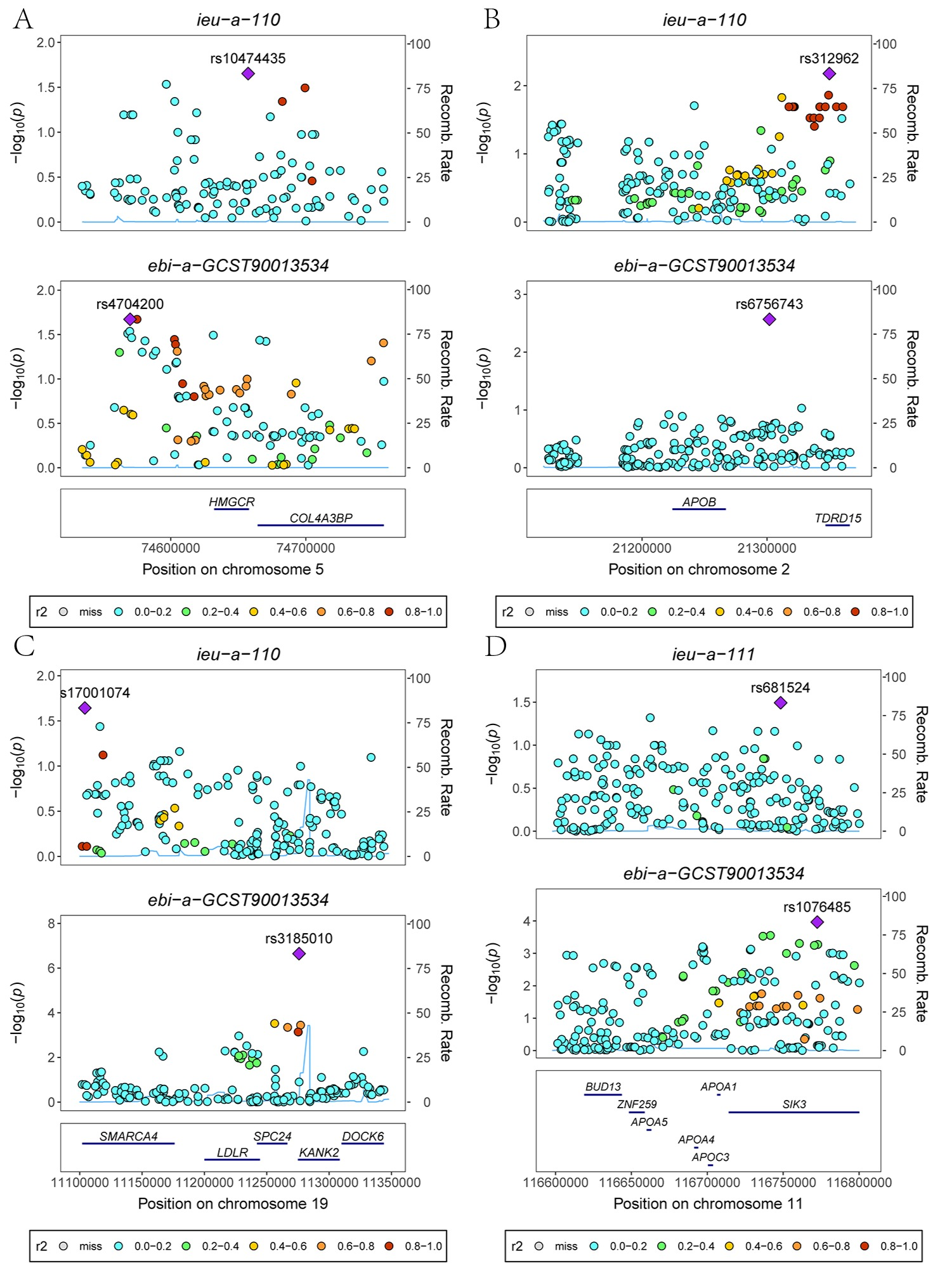

Supplement: S5 Fig — A. Results of co-localization analysis of HMGCR-mediated LDL-C and RA; B. Results of co-localization analysis of APOB-mediated LDL-C and RA; C. Results of co-localization analysis of LDLR-mediated LDL-C and RA; D. Results of co-localization analysis of APOC3-mediated TG and RA. (TIF) [file pone.0298629.s006.tif]
